# A novel homozygous intronic variant affecting splicing in the *RYR1* gene contributes to fetal hydrops

**DOI:** 10.1016/j.gendis.2024.101375

**Published:** 2024-07-14

**Authors:** Wei Hou, Guifang Huang, Hongyu Wei, Wenwei Li, Houfeng Huang, Yuling Qiu, Hengying Zhu, Huifeng Han, Ping Chen, Xue Zhang

**Affiliations:** a Collaborative Innovation Centre of Regenerative Medicine and Medical BioResource Development and Application Co-constructed by the Province and Ministry, Guangxi Medical University, Nanning, Guangxi 530021, China; b NHC Key Laboratory of Thalassemia Medicine, Nanning, Guangxi 530021, China; c Guangxi Key Laboratory of Thalassemia Research, Life Sciences Institute, Guangxi Medical University, Nanning, Guangxi 530021, China; d Department of Eugenic Genetics, Baise Maternal and Child Health Hospital, Baise, Guangxi 533000, China; e Department of Organic Chemistry and Medicinal Chemistry, Pharmaceutical College, Guangxi Medical University, Nanning, Guangxi 530021, China; f Department of Ultrasound Imaging, Baise Maternal and Child Health Hospital, Baise, Guangxi 533000, China; g Department of Bioinformatics, Berry Genomics Co., Ltd., Beijing 102206, China; h McKusick-Zhang Center for Genetic Medicine, Institute of Basic Medical Sciences Chinese Academy of Medical Sciences, School of Basic Medicine Peking Union Medical College, Beijing 100730, China

Fetal hydrops is a rare but serious fetal developmental abnormality characterized by the abnormal accumulation of large amounts of fluid in the fetus resulting in generalized edema, and clinically manifested by abnormal functioning of multiple organs and systems.^1^ The ryanodine receptor 1 (*RYR1*) gene encodes the ryanodine receptor found in skeletal muscle and is expressed predominantly in cardiac and skeletal muscle.^2^ The encoded protein functions as a calcium release channel in the sarcoplasmic reticulum, but is also used to connect the sarcoplasmic reticulum to the transverse tubules. Mutations in this gene have been associated with malignant hyperthermia susceptibility, central core disease, and micro-nucleated cardiomyopathy with extraocular muscle paralysis.^3^ Existing studies have also found that mutations in the *RYR1* gene, as well as mutation-induced purebred shear variants, can be associated with fetal hydrops.^4^^,^^5^ Here, we report a novel heterozygote intronic variant affecting *RYR1* gene splicing may cause fetal hydrops.

In this study, we studied a pedigree with a history of 3 cases of hydrops fetalis. The couple in this pedigree were healthy, had a non-consanguineous marriage, and had normal results for thalassemia screening. The mother was blood group AB and Rh positive. They had neither a family history of neuromuscular disease nor malignant hyperthermia, and no other examinations were performed. The obstetric history of the couple revealed a total of six pregnancies, of which two were live births (II2, II6), one abortion (II1), and three hydropic fetuses (II3, II4, II5) (Fig. 1A, B).

**Figure 1:**
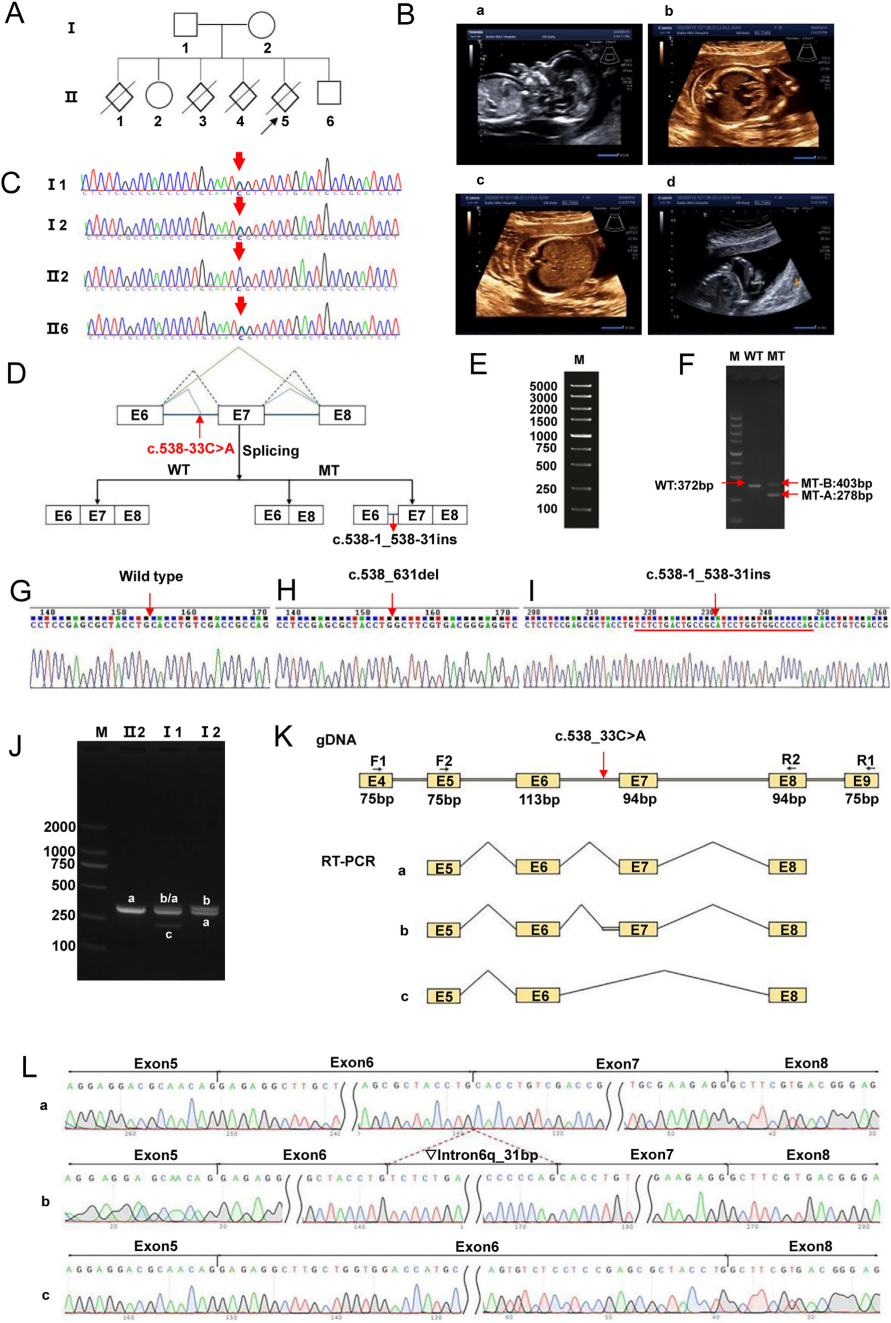
The c.538-33C > A homozygous mutation of the RYR1 gene may be a causative factor in hydatidiform fetuses. **(A)** Family pedigree. **(B)** Ultrasound imaging of the hydrops fetalis. **(a)** At 12^+4^ weeks of amenorrhea, the image of the edematous fetus for Ⅱ5 depicted widespread fetal edema, with a nuchal translucency (NT) thickness of 7.22 mm and abdominal fluid accumulation. **(b**–**d)** At 17^+5^ weeks of amenorrhea, the image of the edematous fetus for Ⅱ5 revealed generalized skin edema, bilateral pleural effusions, fixed hand posture, and partial scoliosis of the spine. **(C)** Sanger sequencing chromatogram of the familial RYR1 gene c.538-33C > A mutation. The variation was identified as heterozygous in Ⅰ1 (father), Ⅰ2 (mother), and Ⅱ6 (their son), while Ⅱ2 (their daughter) was identified as homozygous wild-type. Arrows represent the variant. **(D)** Schematic representation of the splicing pattern. The splice diagram illustrated that this mutation resulted in splicing variations in the RYR1 gene. **(E–I)** The splicing pattern of the c.538-33C > A mutation in the RYR1 gene was validated through minigene experiments. WT and MT (c.538-33C > A) plasmids of the gene were transfected into 293T cells. RNA was extracted, reverse transcribed into cDNA, and then subjected to PCR. By analyzing the size of the PCR fragments and PCR sequencing results, abnormal splicing of the MT mRNA was identified. The bands corresponding to WT plasmids represent correct splicing, the MT-A band represents the complete deletion of exon 7, and the MT-B band represents the insertion of a 31-bp sequence in the sixth intron of RYR1. **(J)** Agarose gel electrophoresis of reverse transcription PCR products. Ⅱ2 displayed a single band, labeled as band a; Ⅰ1 exhibited three bands, labeled from largest to smallest as band b, band a, and band c; Ⅰ2 showed two bands, with the larger band labeled as band b, and the smaller band labeled as band a. **(K)** Schematic diagram of primer design and splicing, with the mutation position indicated by the red arrow. **(L)** Sequencing results corresponding to splicing bands a, b, and c. Band a represents normal splicing, with the splicing pattern as Exon5 (79bp)-Exon6 (113bp)-Exon7 (94bp)-Exon8 (94bp); band b represents abnormal splicing with intron 6 retention of 31 bp, with the splicing pattern as Exon5 (79bp)-Exon6 (113bp)-▽Intron6 (31bp)-Exon7 (94bp)-Exon8 (94bp); band c represents abnormal splicing with exon 7 skipping, with the splicing pattern as Exon5 (79bp)-Exon6 (113bp)-Exon8 (94bp).

To identify the causative genes in the 3rd hydrops fetuses, we performed whole exome sequencing on the umbilical cords of the hydrops fetuses of II4 and II5 pregnancies and the peripheral blood of the parents and identified a new shear variant mutation c.538-33C > A in intron 6 of the *RYR1* gene, which belongs to the deep intronic mutation region affecting shear. Both II4 and II5 were heterozygous for this mutation; the parents were heterozygous, II2 was wild-type, and II6 was heterozygous. Sanger sequencing results verified this result (Fig. 1C). The research obtained approval from the Ethics Committee of Guangxi Medical University (No. 20240159), and all participants involved in the study provided written informed consent.

The c.538-33C > A mutation in the *RYR1* gene is autosomal recessive and is categorized as a variant of uncertain significance according to the ACMG (American College of Medical Genetics and Genomics) guidelines. RDDC (Rare Disease Data Center)^SC^, HSF (Human Splicing Finder), and SpliceAI shear analyses showed that the mutation may be consistent with the wild-type shear pattern or lead to effects such as intron lagging or exon jumping.

To further characterize the effect of the mutation at the mRNA transcription level, we first performed *in vitro* minigene splicing experiments. Seamless cloning was used to design primers (*RYR1*-F: AAGCTTGGTACCGAGCTCGGATCCGAGAGGCTTGCTGGTGGACCATGCACCC, *RYR1*-R: TTAAACGGGCCCTCTAGACTCGAGCTGCGCTGGGTCATCACTGTCAGCAGGGGG) that were amplified from normal and genomic DNA carrying the NM_000540.3: c.538-33C > A mutation locus to construct the *RYR1* wild-type minigene plasmid (WT) and the *RYR1*(c.538-33C > A) mutant minigene plasmid (MT), respectively, then inserted into pMini-CopGFP vector, and then transfected into HEK-293T cells. Total RNA was extracted by Trizol and then cDNA was reverse transcribed. cDNA was amplified by reverse transcription PCR with designed primers (MiniRT-F: GGCTAACTAGAGAACCCACTGCTTA, *RYR1*-RT-R: CTGCGCTGGGTCATCACTGTCAGC). cDNA was amplified by analyzing the size of the PCR fragments and PCR sequencing results to identify the aberrant splicing of the mutant mRNA. We found that the post-transcriptional mRNA sequence of the wild-type plasmid was as expected, containing a complete mRNA product transcribed from exon 6, exon 7, and exon 8. In contrast, the sequence of the mutant plasmid shows that the c.538-33C > A mutation results in two forms of variant splicing, the first being a complete deletion of exon 7, with the mRNA expressed as c.538_ 631delCACCTGTCGACCGCCAGTGGGGGAGCTCCAGGTTGACGCTTCCTTCATGCAGACACTATGGAACATGAACCCCATCTGCTCCCGCTGCGAAGAGG; and the second was the retention of a 31 bp sequence in intron 6, and the mRNA was expressed as c.538-1_538-31insTCTCTGACTGCCGCATCCTGGTGGGCCCCCAG. (Fig. 1D–I).

In addition, *in vivo* reverse transcription PCR splicing analysis was performed in this study. Total RNA was extracted from parents and II2, reverse transcribed into cDNA as a template, and amplified using designed reverse transcription PCR primers (*RYR1*-F1: CTCCTGTATGGCCATGCCAT, *RYR1*-F2: GCTCCATGACTGACAAGCTG, *RYR1*-R2: CAGGGGAAATGGGTCAGACAC. *RYR1*-R1: TGATTCTCAGTGGCTCCAGC). Then it was analyzed by agarose gel electrophoresis and sequencing. We found that mutation c.538-33C > A affected the normal splicing of mRNA, and the mutation resulted in a 31 bp stall on the right side of intron6 or an exon 7 jump. II 2 was spliced normally. The father (I1) had normal splicing and two abnormal splicing bands, right intron 6 retention of 31 bp, and exon 7 skipping. The mother (I2) was spliced normally and intron 6 retained an abnormal splice band of 31 bp (Fig. 1J–L). Intron6 right side retention of 31 bp was represented at the cDNA and protein level as c.537_538insTCTCTGACTGCCGCATCCTGGTGGGCCCCCAG p.His180Serfs∗22, and the mutation caused a premature termination in the exon 7 and produced the early termination codon PTC, resulting in a subsequent reading frame change that would produce a truncated protein of 200aa in length. The exon 7 jump was represented at the cDNA and protein level as c.538_631del p.His180Alafs∗3, and the mutation produced the early termination codon PTC in exon 8, resulting in subsequent reading frame alterations that would produce a truncated protein of length 181aa.

In summary, the pedigree with parents heterozygous for c.538-33C > A of the *RYR1* gene did not develop hydrops, whereas the two hydrops fetuses examined were both heterozygous; therefore, we suggest that the c.538-33C > A heterozygous mutation of the *RYR1* gene, which affects the normal splicing of the mRNA, may contribute to the development of hydrops in the fetus. This is the first report of this mutation and provides a novel mechanism for fetal hydrops. However, in this study, we only focused on the shearing variant caused by the *RYR1* gene mutation, and there may be other factors involved in the mechanism of fetal hydrops, and we also need further studies to validate these findings to develop a more comprehensive and accurate therapeutic regimen.

## Author contributions

**Wei Hou:** Conceptualization, Data curation, Investigation, Methodology, Resources, Validation, Writing – original draft, Writing – review & editing. **Guifang Huang:** Data curation, Investigation, Methodology, Resources. **Hongyu Wei:** Investigation, Methodology, Validation. **Wenwei Li:** Investigation, Methodology, Validation. **Houfeng Huang:** Data curation, Investigation, Methodology. **Yuling Qiu:** Investigation, Methodology, Validation. **Hengying Zhu:** Investigation, Validation. **Huifeng Han:** Data curation, Methodology. **Ping Chen:** Conceptualization, Data curation, Funding acquisition, Investigation, Methodology, Supervision, Writing – review & editing. **Xue Zhang:** Conceptualization, Data curation, Formal analysis, Funding acquisition, Methodology, Project administration, Resources, Supervision, Writing – original draft, Writing – review & editing.

## Conflict of interests

The author declared no conflict of interests.

## Funding

This research was funded by the Guangxi Science and Technology Department (China) (No. Guike AD23026025).
