# Supplementary material for: A novel homozygous intronic variant affecting splicing in the RYR1 gene contributes to fetal hydrops
Source: Genes Dis. 2024 Jul 14;11(6):101375. doi: 10.1016/j.gendis.2024.101375 (PMC11359738; doi:10.1016/j.gendis.2024.101375)
Supplement: Multimedia component 1 [file mmc1.docx]

**Material and methods**

***Ethical compliance and patient’s information***

The couple were healthy, and non consanguineous marriage. They had no thalassemia. The wife's blood type was AB type with Rh-positive. They had no history of neuromuscular disease and malignant hyperthermia, or family history was found. Other findings were not checked. The obstetric history of this couple reveals a total of six pregnancies, resulting in two live births(Ⅱ2,Ⅱ6), one induced abortion(Ⅱ1), and three cases of hydrops fetalis(Ⅱ3,Ⅱ4,Ⅱ5).

In 2017, the fetus (Ⅱ3)conceived was terminated at 12 weeks of gestation due to fetal hydrops detected by ultrasound, but no further examinations were conducted.

In 2019, during the 13th week of gestation, the fetus (II4) conceived exhibited general hydrops detected by ultrasound, leading to termination through a surgical procedure. Cord blood was obtained from the fetus for chromosomal copy number variation (CNV) testing, which yielded no abnormalities. Both partners underwent peripheral blood chromosome testing and with normal results.

In 2020, at 12 weeks and 4 days of gestation, ultrasound examination revealed that the fetus (II5) measured equivalent to 13+ weeks, exhibited general hydrops, a nuchal translucency (NT) thickening of 7.22mm, and abdominal fluid accumulation. Pre-natal diagnosis through chorionic villus sampling identified the presence of 3.8Mb and 19.9Mb of absence of heterozygosity (AOH) in the 1p33p32.3 and 19p12q13.12 regions, with unclear clinical significance. Chromosomal karyotyping showed no abnormalities. At the gestational age of 17 weeks and 5 days, ultrasound indicated that the fetus measured equivalent to 18 weeks and 1 day, presenting with generalized edema of the fetal trunk skin, bilateral pleural effusion, fixed hand posture, and partial spinal scoliosis. Pregnant women choose to terminate their pregnancies.

In this research, Umbilical cord samples from the edematous fetuses of Ⅱ4 andⅡ5, along with peripheral blood samples from their parents, were subjected to whole-exome sequencing analysis. The research obtained approval from the Ethics Committee of Guangxi Medical University, and all participants involved in the study provided written informed consent.

***Library Construction and Sequencing***

DNA samples were fragmented into approximately 200-bp fragments using 50 ng of DNA. Subsequently, end repair and 3’-end adenylation were performed. The DNA fragments were ligated to sequencing junctions containing the barcode sequence. The ligation product was purified without the need for PCR. Liquid-phase hybridization was performed on a pre-library following the standard protocol for NanoWES chips. Hybridization products were eluted and collected, followed by PCR amplification and purification in order to produce exon libraries. Subsequently, the libraries were quantified using qPCR.

High-throughput PE150 sequencing was performed on an Illumina Novaseq6000 platform (Illumina, San Diego, CA, USA). Raw data were processed using CASAVA v1.82. Base quality criteria were set at a minimum of 85% of bases with a Phred quality score (Q) of 30 or above (≥Q30), minimum of 95% of bases with a Q value of 20 or above (≥Q20), and duplication rate of not more than 30%.

***Data Analysis***

The genomic sequencing raw data were subjected to quality control filtering and aligned to the human reference genome (hg19/GRCh37) using the Burrows-Wheeler Aligner tool,^15^ with removal of PCR repetitive sequences using Picard v1.57 (http://picard.sourceforge.net/). Variant detection analysis was performed using Verita Trekker variant detection system and GATK (https://software.broadinstitute.org/gatk/). Variant annotation was performed using ANNOVAR and the Enliven Variant Annotation Interpretation System developed by Berry Genetics.^16^

Interpretation and categorization of reports were performed according to the American College of Medical Genetics and Genomics (ACMG) guidelines for genetic variant interpretation.^17^ Variants with a minimum allele frequency (MAF) <0.5% or affected by splicing in exon regions were subjected to in-depth interpretation based on the types of ACMG, evidence of pathogenicity, clinical summaries of associated disorders, and genetic models.

***Sanger sequencing***

Blood samples from both spouses, as well as from Ⅱ2, and amniotic fluid from Ⅱ6 were subjected to Sanger sequencing verification. The validation primers were as follows: *RYR1*_1F:5'-AGAACATCCACGAGCTCTGG-3', *RYR1*_1R：5'-CCAGACTATGACCCCTGACC-3'). The sequencing was conducted by Beijing Berry and Kang Medical Laboratory Co., Ltd.

***Splice prediction analysis***

The mutations were subjected to splice prediction analysis using RDDCSC (https://rddc.tsinghua-gd.org/search-middle?to=SplitToolModel), SpliceAI (https://spliceailookup.broadinstitute.org/), and HSF (http://www.umd.be/HSF3/HSF.shtml).

***Construction and Splicing Analysis of Minigene***

Primers (*RYR1*-F: 5'-AAGCTTGGTACCGAGCTCGGATCCGAGAGGCTTGCTGGTGGA

CCATGCACCC-3', *RYR1*-R: 5'-TTAAACGGGCCCTCTAGACTCGAGCTGCGCTGGTCATCA

CTGTCAGCAGGGG-3') were designed for seamless cloning to amplify genomic DNA from a normal individual and an individual carrying the NM_000540.3: c.538-33C>A mutation site, yielding two target-inserted gene fragments.

The plasmid insertion sequence was designed to include three exons and two introns, and wild-type (WT) *RYR1* minigene plasmid and mutant (MT) *RYR1* (c.538-33C>A) minigene plasmids were constructed separately. These were then inserted into the pMini-CopGFP vector and transfection into HEK-293T cells. We utilized TRIzol to extract total RNA, and reverse transcription of RNA was performed to obtain cDNA. The cDNA was amplified through RT-PCR using the specified primers (MiniRT-F: 5'-GGCTAACTAGAGAACCCACTGCTTA-3', MiniRT-R: 5'-CTGCGCTGGTCATCACTGTCAGC-3'). Subsequently, the resulting amplification products were analyzed through sequencing and agarose gel electrophoresis to draw experimental conclusions.

***In vivo RT-PCR splicing analysis***

The peripheral blood of both spouses and Ⅱ2 was extracted using a whole-blood RNA extraction kit (TIANMO, TR121-50) to obtain total RNA, which was then reverse transcribed into cDNA. The designed primers (*RYR1*-F1: 5′-CTCCTGTATGGCCATGCCAT-3′, *RYR1*-R1: 5′-TGATTCTCAGTGGCTCCAGC-3′) were used for PCR amplification with the reverse-transcribed cDNA as a template. The amplified products were subsequently subjected to further amplification using primers (*RYR1*-F2: 5′-GCTCCATGACTGACAAGCTG-3′, *RYR1*-R2: 5′-CAGGGGAAATGGTCAGACAC-3′) and analyzed through agarose gel electrophoresis. After purifying the PCR products, Sanger sequencing was carried out.
